# Efficacy and safety of artemether-lumefantrine for the treatment of uncomplicated falciparum malaria in mainland Tanzania, 2019

**DOI:** 10.1186/s12936-024-04931-0

**Published:** 2024-04-09

**Authors:** Billy E. Ngasala, Mercy G. Chiduo, Bruno P. Mmbando, Filbert T. Francis, Samwel Bushukatale, Twilumba Makene, Celine I. Mandara, Deus S. Ishengoma, Erasmus Kamugisha, Maimuna Ahmed, Muhidin K. Mahende, Reginald A. Kavishe, Florida Muro, Fabrizio Molteni, Erik Reaves, Chonge Kitojo, George Greer, Ssanyu Nyinondi, Bilal Kabula, Shabbir Lalji, Frank Chacky, Ritha J. Njau, Marian Warsame, Ally Mohamed

**Affiliations:** 1 Department of Parasitology, School of Public Health, Muhimbili University of Health and Allied Sciences, Dar es Salaam, Tanzania; 2 National Institute for Medical Research, Tanga Research Centre, Tanga, Tanzania; 3 National Institute for Medical Research, Headquarters, Dar es Salaam, Tanzania; 4 Catholic University of Health and Allied Sciences, Bugando Medical Centre, Mwanza, Tanzania; 5 Ifakara Health Institute, Dar-es-Salaam, Tanzania; 6 Kilimanjaro Christian Medical Centre, Kilimanjaro Christian Medical University College, Moshi, Tanzania; 7 National Malaria Control Program, Dodoma, Tanzania; 8 U.S. President’s Malaria Initiative, U.S. Centers for Disease Control and Prevention, Dar es Salaam, Tanzania; 9 U.S. President’s Malaria Initiative, U.S. Agency for International Development, Dar es Salaam, Tanzania; 10 R.T.I. International, Dar es Salaam, Tanzania; 11 Muhimbili University of Health and Allied Science, Dar es Salaam, Tanzania; 12 Gothenburg University, Gothenburg, Sweden

**Keywords:** Malaria, *Plasmodium falciparum*, Artemether-Lumefantrine, Therapeutic efficacy

## Abstract

**Background:**

Artemisinin-based combination therapy (ACT) has been a major contributor to the substantial reductions in global malaria morbidity and mortality over the last decade. In Tanzania, artemether-lumefantrine (AL) was introduced as the first-line treatment for uncomplicated *Plasmodium falciparum* malaria in 2006. The World Health Organization (WHO) recommends regular assessment and monitoring of the efficacy of the first-line treatment, specifically considering that artemisinin resistance has been confirmed in the Greater Mekong sub-region. This study's main aim was to assess the efficacy and safety of AL for treating uncomplicated *P. falciparum* malaria in Tanzania.

**Methods:**

This was a single-arm prospective antimalarial drug efficacy trial conducted in four of the eight National Malaria Control Programme (NMCP) sentinel sites in 2019. The trial was carried out in outpatient health facilities in Karume-Mwanza region, Ipinda-Mbeya region, Simbo-Tabora region, and Nagaga-Mtwara region. Children aged six months to 10 years with microscopy confirmed uncomplicated *P. falciparum* malaria who met the inclusion criteria were recruited based on the WHO protocol. The children received AL (a 6-dose regimen of AL twice daily for three days). Clinical and parasitological parameters were monitored during follow-up over 28 days to evaluate drug efficacy.

**Results:**

A total of 628 children were screened for uncomplicated malaria, and 349 (55.6%) were enrolled between May and September 2019. Of the enrolled children, 343 (98.3%) completed the 28-day follow-up or attained the treatment outcomes. There were no early treatment failures; recurrent infections during follow-up were common at two sites (Karume 29.5%; Simbo 18.2%). PCR-corrected adequate clinical and parasitological response (ACPR) by survival analysis to AL on day 28 of follow-up varied from 97.7% at Karume to 100% at Ipinda and Nagaga sites. The commonly reported adverse events were cough, skin pallor, and abdominal pain. The drug was well tolerated, and no serious adverse event was reported.

**Conclusion:**

This study showed that AL had adequate efficacy and safety for the treatment of uncomplicated falciparum malaria in Tanzania in 2019. The high recurrent infections were mainly due to new infections, highlighting the potential role of introducing alternative artemisinin-based combinations that offer improved post-treatment prophylaxis, such as artesunate-amodiaquine (ASAQ).

**Supplementary Information:**

The online version contains supplementary material available at 10.1186/s12936-024-04931-0.

## Background

Artemisinin-based combination therapy (ACT) has been a major contributor to the substantial reductions in global malaria morbidity and mortality over the last decade [1]. The most commonly used artemisinin-based combinations in Africa are artemether-lumefantrine (AL) and artesunate-amodiaquine (ASAQ) [2]. AL was introduced as the first-line treatment for uncomplicated *Plasmodium falciparum* malaria in 2006 in Tanzania. Continuous use of a single ACT may result in unidirectional selection of resistant parasites [3, 4].

Despite the high ACT cure rates observed in Africa, studies conducted in Tanzania and other parts of Africa provide evidence for in vivo selection of lumefantrine tolerant/resistant parasites [5, 6]. Tolerance to AL has been linked to the selection of single nucleotide polymorphism (SNPs) associated with parasite drug tolerance/resistance in the *P. falciparum* multidrug resistance gene 1 (*pfmdr1*) at N86Y, Y184F and D1246Y; *P. falciparum* chloroquine resistance transporter gene (*pfcrt*) at K76T; and *P. falciparum* multidrug resistance-associated protein gene (*pfmrp1*) [7, 8]. Importantly, no clear evidence of increased *pfmdr1* gene copy, previously linked to lumefantrine resistance in South-East Asia, exists to date in East Africa. After treatment with AL, lumefantrine selects for *pfmdr1* N86, 184F, D1246, and *pfcrt* K76 [7, 8]. This hypothesis is supported by data from Bagamoyo District, indicating that reinfecting *P. falciparum* parasites harboring the *pfmdr1* N86/184F/D1246 haplotype were able to withstand 15-fold higher lumefantrine blood concentrations than those with the alternative haplotype (86Y/Y184/1246Y) [6, 9].

The development of tolerance/resistance against the long-acting partner drugs in ACT, such as lumefantrine and amodiaquine, has been suggested to start through post-treatment selection among recurrent infections of less sensitive *P. falciparum* parasites as reinfecting parasites need to be able to survive the exposure of sub-therapeutic blood levels of the long-acting drug and its active metabolites. This may, in turn, lead to a gradually shortened post-treatment prophylactic period, long before clinical treatment failures are apparent, which is why temporal surveillance of efficacy and genetic anti-malarial drug resistance markers of *P. falciparum* has been proposed as an early warning system of evolution of ACT tolerance/resistance [10, 11].

Furthermore, gains in elimination efforts are threatened by the recent emergence of artemisinin and partner drug resistance in Southeast Asia [12]. This region has been the epicentre for the evolution and spread of resistance to every region. Evidence of reduced susceptibility to artemisinin in Western Cambodia was first reported in January 2007 and confirmed in subsequent detailed studies [3, 12, 13]. A major concern is that artemisinin and partner drug resistance may spread across a wider geographic area, as chloroquine resistance did in the 1960s and 1970s, moving from Southeast Asia to the Indian subcontinent and subsequently to Africa, which bears the vast majority of the global malaria burden. Partial artemisinin resistance has now been detected in several sub-Saharan African countries, including Uganda, Eritrea, and Rwanda [3, 4, 14, 15].

The development of partial resistance to artemisinin in Uganda, Eritrea, and Rwanda did not originate from the spread of genetic mutations from the Greater Mekong Subregion (GMS) but arose de novo [16]. Recently, partial artemisinin resistance was detected in the Kagera region of Tanzania [17]. The study revealed that 17% of patients in Kagera exhibited day three parasitaemia. Additionally, 10% of patients showed both day three parasitaemia and a WHO-validated *k13* mutation (R561H), suggesting the presence of partial artemisinin resistance.

The National Malaria Control Programme (NMCP) in collaboration with its implementing partners conducts therapeutic and safety studies at selected sentinel sites at least every two years as recommended by the World Health Organization (WHO) [18]. Recent AL studies have shown a high cure rate of > 95% [5, 18–20]. This study was conducted to provide updated information on the AL’s efficacy and safety for treating uncomplicated *P. falciparum* malaria in Tanzania.

## Methods

### Study design

This was a single-arm prospective study for assessing the therapeutic efficacy and safety of AL for the treatment of uncomplicated falciparum malaria in children aged between six months and 10 years.

### Study sites

**Fig. 1:**
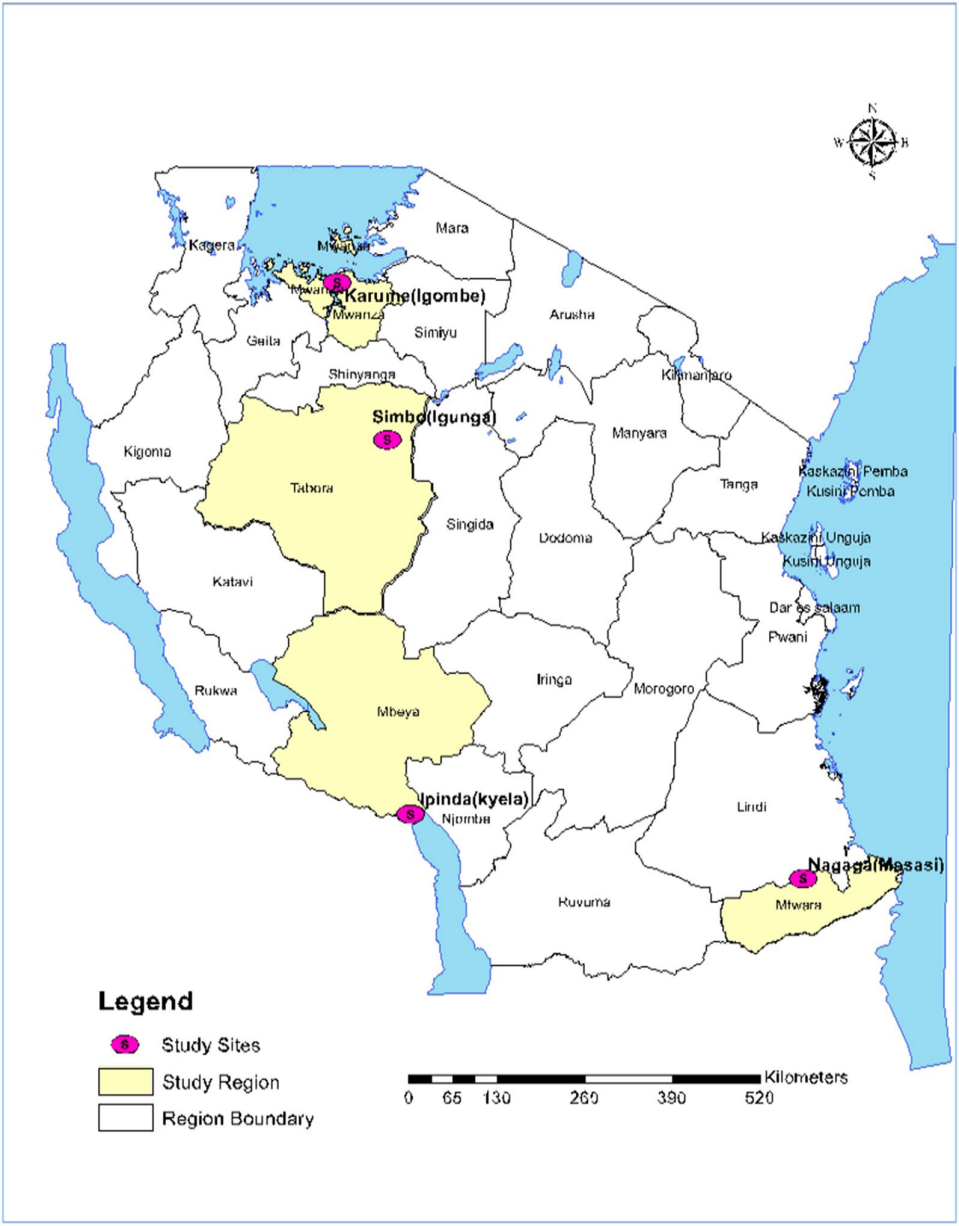
The four study sites for antimalarial therapeutic efficacy in Tanzania in 2019

The study was conducted at four of the eight NMCP sentinel sites (Karume-Mwanza region, Ipinda-Mbeya region, Simbo-Tabora region, and Nagaga-Mtwara region) between May and September 2019. The study sites (Fig. 1) have been NMCP sentinel sites for monitoring of antimalarial efficacy since 1997 [5, 20, 21]. The eight primary or secondary health care facilities included in Fig. 1 represent basic features of geographic zones for mainland Tanzania. This geographical diversity has been considered as representative of the malaria epidemiology in Tanzania based on the past and current shift of malaria transmission intensity [22]. Malaria transmission in the majority of areas in Mainland Tanzania tends to be characterized as low to moderate [22]. However, seasonal peaks of malaria transmission occur subsequent to the primary long rainfall season, typically between March and June, although the specific timing may vary depending on the patterns of rainfall. Throughout Tanzania, *P. falciparum* is the predominant malaria species and *Anopheles gambiae *sensu stricto (*s.s.*)*, Anopheles arabiensis,* and *Anopheles funestus* are now the primary vectors of human malaria in East Africa [23, 24].

### Study population

Children aged between six months and 10 years presenting with fever (axillary temperature ≥ 37.5 °C and/or reported history of fever in the past 24 h) were screened for possible enrollment into the study following inclusion and exclusion criteria as follows: mono-infection of *P. falciparum* detected by microscopy, parasitaemia between 250 and 200,000 asexual parasites/μl of blood, ability to swallow oral medications, ability and willingness to attend scheduled follow-up visits, informed consent provided by parent or guardian, and stable residence within the catchment area throughout the study period. Exclusion criteria included patients with negative malaria rapid diagnostic test (RDT) results and general danger signs or signs of severe malaria. Danger signs of malaria in children consisted of the following clinical presentation: child unable to drink or breastfeed, vomiting, recent history of convulsions, lethargic or unconscious state, unable to sit or stand, and difficulty in breathing [18]. Patients with mixed or mono-infections with another *Plasmodium* species, severe anaemia (Hb < 5 g/dL), or presence of severe malnutrition (defined as a child who had symmetrical edema involving at least the feet or mid-upper arm circumference < 110 mm) were excluded from the study. Other exclusion criteria included febrile conditions due to diseases other than malaria (e.g., measles, acute lower respiratory tract infection, severe diarrhoea with dehydration) or other known underlying chronic or severe diseases (including cardiac, renal, and hepatic diseases, and HIV/AIDS)**,** regular medications that may interfere with anti-malarial pharmacokinetics, and history of hypersensitivity reactions or contraindications to AL. Excluded patients received appropriate treatment according to the national guidelines [25].

### Sample size estimation

The sample size was determined based on the WHO 2009 standard protocol with the assumption that 5% of the patients treated with AL were likely to have treatment failure [18]. At a confidence level of 95% and an estimated precision of 5%, the minimum sample size was 73 patients at each site. With a 20% increase to allow for loss to follow-up and withdrawals during the 28-day follow-up period, 88 patients were targeted per site.

### Sample collection

Blood samples were collected through a finger prick for malaria RDT (Carestart™ ACCESSBIO, USA) and thick and thin blood smears for detection of malaria parasites by microscopy. From each patient, dried blood spots (DBS) on Whatman III filter papers were collected for laboratory analysis of malaria parasites, including *P. falciparum* diversity, molecular markers of antimalarial resistance, and distinguishing recrudescent from new infections by PCR genotyping. Thick blood smears were stained with 3% Giemsa for 30–45 min and examined by microscopy to detect presence of malaria parasites and the level of parasitemia. Parasitaemia was measured by counting the number of asexual parasites against 200 white blood cells (WBCs) in thick blood films; thin blood films were examined for detection of the different parasite species as previously described [20]. A thick blood smear was declared negative when examination of 100 high power fields did not reveal the presence of any malaria parasite. For quality control, each slide was re-examined by a second microscopist and those with discrepancy were re-examined by a third microscopist. Further disagreement was resolved by a team of three microscopists who examined the same slide at the same time. Final parasitaemia was calculated as the average between the two closest readings.

### Patient treatment and follow-up

During screening, patients were clinically examined before going to the laboratory for sample collection. Patients enrolled in the study were treated with AL (Coartem®, Beijing Novartis Pharma Ltd, Beijing China for Novartis Pharma AG, Basle, Switzerland, obtained from the WHO). This was a fixed dose combination of 20 mg of artemether and 120 mg lumefantrine in a tablet. The drugs were administered without food according to the recommended doses based on the weight of patient [25]. While treatment did not include the provision of food, caregivers were advised to supply fatty meals at home to enhance the drug's absorption. Patients were monitored for 30 min to confirm the absence of any vomiting related to the study drugs. One tablet was given to children weighing 5–14 kg; two tablets to children weighing 15–24 kg, and three tablets to children weighing 25–35 kg. A full course of AL consisted of 6-doses given twice daily (8 hourly apart on day 0 and 12 h apart on days 1 and 2). The study drugs were given under direct supervision of the study nurses at the health facility. In case of failure, the participants were treated following the National Malaria Treatment Guidelines [25].

All enrolled patients were followed for 28 days with scheduled visits on days 1, 2, 3, 7, 14, 21, and 28. During follow-up visits, clinical and safety assessments were performed, axillary temperature was measured, and a blood slide for parasite count was taken. On day 7, 14, and 28, DBS were collected on filter papers for genotyping. The patients and their parents or guardians were also informed to return on any day if the symptoms returned or any other danger signs were present. Patients who could not come for their scheduled visit by mid-day were visited at home by a member of the study team and asked to come to the health facility. In case a patient travelled outside the study area and could not be traced for scheduled follow-up within 2 days, he/she was withdrawn from the study. Patients who did not attend the scheduled visits or could not be found despite all reasonable efforts were classified as lost to follow-up.

### Sample processing and analysis

Parasite DNA was extracted from DBS using QIAamp DNA blood Midi Kit (Qiagen GmbH, Hilden, Germany) according to the manufacturer’s instructions. All paired samples collected on day 0 and day of recurrent infection were genotyped by utilizing merozoite surface proteins 1 and 2 (msp1 and msp2) and glutamate rich protein (glurp) using gel electrophoresis. Bands were considered a match if the day 0 and day of failure fragment lengths were within 10 base pairs for msp1 and msp2 and within 50 base pairs for glurp.

Reinfection and recrudescence were differentiated using both the 3/3 and 2/3 methods as recommended by the WHO [26]. The 3/3 algorithm required at least one matching band in any sub-allele for all three makers. If Day 0 and recurrent samples shared alleles for at least 2/3 markers it was classified as recrudescence. If insufficient matches were identified, the recurrence was classified as reinfection. If there were no amplification products resulting in sharp, defined bands in both the day 0 and day of failure samples for a gene, that gene was not used to distinguish between recrudescence and reinfection. Determinations using the 3/3 method were used for analysis; 2/3 results were added in the supplemental materials [26, 27]. Raw genotyping data have been included as supplemental material (Additional file 1: Table S1).

The following molecular markers were genotyped to assess for anti-malarial drug resistance: multidrug resistance 1 (*mdr1*) and polymorphisms in kelch propeller domain (*k13*) [13] by capillary sequencing, and *mdr1* copy number variants according to published protocols^27^. SNPs calling in *mdr1* and *k13* was performed using Geneious® analysis software (Biomatters, New Zealand; www.geneious.com) by mapping the sequence data on the 3D7 reference sequences. Raw sequence reads were cleaned using default settings and reads with high-quality scores (the percentage of high-quality bases) below 70% were discarded from further analysis. The *pfk13* (codon positions 440–600) and *pfmdr1* (codon positions: 86, 184 and 1246) were analysed for SNPs. SNPs were called only if they fit the following criteria: (1) they were found in both the forward and reverse reads, (2) they had a p-value of < 0.0001 (p-value represents the probability of a sequencing error resulting in observing bases with at least the given sum of qualities), and (3) they had a minimum strand bias p-value of < 0.0005 when exceeding 65% strand bias, as some errors from sequencing machines are more likely to happen on nearby upstream bases.

### Study outcomes

The primary end point was parasitological cure on day 28 as per WHO protocol of 2009 [18], while secondary end points included occurrence and severity of adverse events and molecular markers of drug resistance. Treatment outcomes were classified as: (1) Early treatment failure (ETF) if the patient had presence of parasitaemia and danger signs on day 1, 2 or 3 or persistence of parasitaemia until day 3; (2) Late clinical failure (LCF) was defined as presence of danger signs or severe malaria, or axillary temperature of ≥ 37.5 °C with parasitaemia between days 4 and 28 in a patient who did not qualify as early treatment failure; (3) Late parasitological failure (LPF) if a patient had parasitaemia between days 7 and 28 in the absence of fever or other clinical symptoms, and was not classified as early treatment failure; (4) Adequate clinical and parasitological response (ACPR) in the absence of parasitaemia in a patient who was not classified as early, late clinical, or late parasitological failure; (5) lost to follow-up when despite all reasonable efforts, an enrolled patient did not attend the scheduled visits and could not be found, and thus the patient was withdrawn from the study; and (6) withdrawal when the patient consented to withdrawal, failed to complete treatment, or there was a protocol violation.

### Data management and analysis

Single data entry was performed at the study sites; this was followed by second entry after the end of data collection. The data was entered into a Microsoft Access database, and later validated, cleaned, and analysed using STATA for Windows, version 11 (STATA Corporation, TX-USA). Descriptive statistics such as percentages, mean, median, standard deviation, and range were reported as appropriate. Treatment outcomes were analysed based on per protocol analysis[18]. In the per protocol analysis, patients with new infections, loss to follow up, withdrawal or protocol violations as well as those with indeterminate PCR results, were excluded. In Kaplan–Meier analysis patients were censored with new infections, lost to follow up, withdrawal, or protocol violations. Patients with indeterminate PCR results were excluded from the analysis of PCR-corrected treatment outcome[18]. Baseline characteristics and primary and secondary outcomes were presented descriptively for the four sites. Continuous variables such as parasite density and age among the four sites were compared using t-test (for normally distributed data) or Mann–Whitney U test (a non-parametric test for non-normally distributed data).

### Ethical consideration

Ethical clearance was obtained from the Medical Research Coordinating Committee (MRCC) of the National Institute for Medical Research (NIMR) with reference number NIMR/HQ/R.8c/Vol.I/1149. Permission to conduct the study at the health facilities was sought in writing from the relevant regional and district medical authorities. Oral and written informed consent was obtained from parents or guardians of all eligible patients before they were screened for possible inclusion into the study.

## Results

### Basic characteristics

**Fig. 2:**
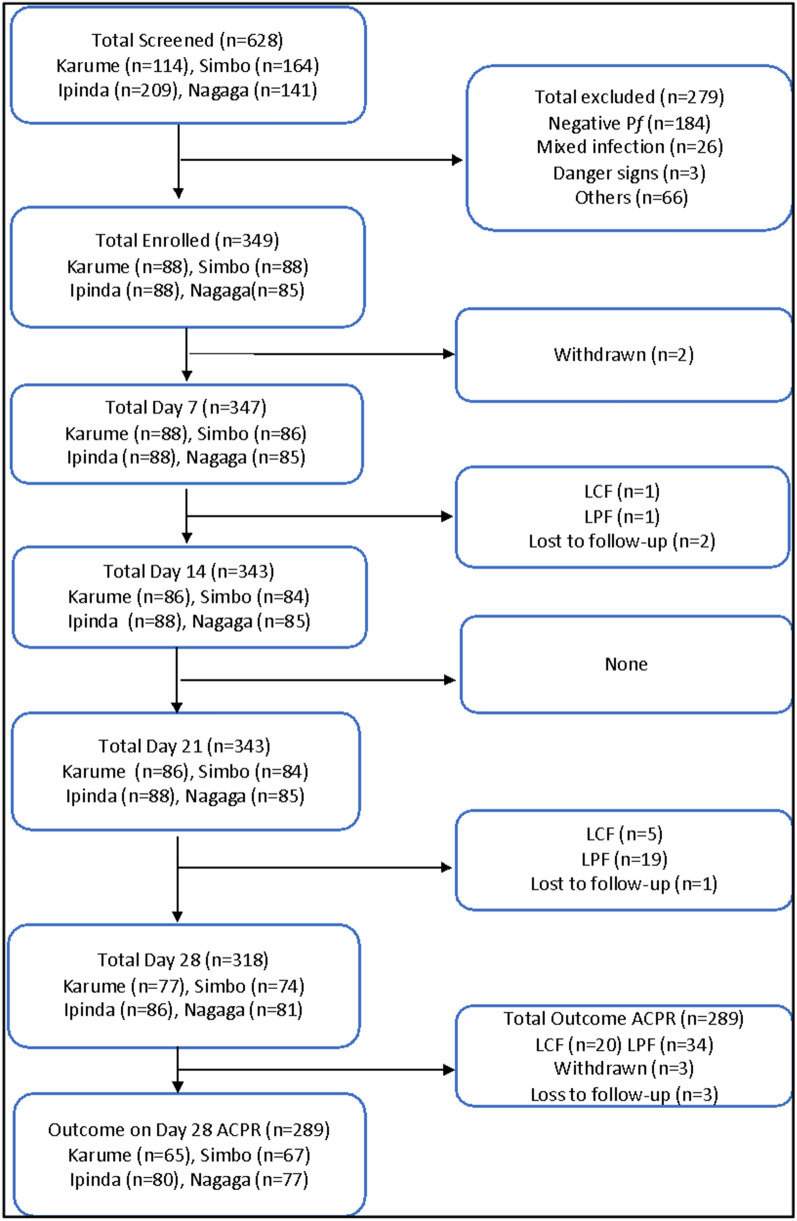
Trial profile showing the flow of patients at screening, enrollment, and follow-up at the four study sites Karume, Ipinda, Simbo, and Nagaga. *ACPR* Adequate clinical and parasitological response, *LCF* Late clinical failure, *LPF* Late parasitological failure

During the study period, 628 children were screened for uncomplicated malaria and 349 (55.6%) were enrolled between May and September 2019. Of the screened children, 279 (44.4%) were excluded because of presence of fever due to other causes (positive RDT but negative blood slide), low parasitaemia outside the defined interval, severe malaria, or living outside the study area (Fig. 2). Only three patients were lost lo follow up (Fig. 2). Table 1 summarizes the demographic and laboratory baseline data of the participants.

**Table 1:** Baseline patient characteristics

Variables	Karume	Simbo	Ipinda	Nagaga
Number screened	114 (18.2%)	164 (26.1%)	209 (33.3%)	141 (22.5%)
Number enrolled	88	88	88	85
Sex (%)				
Female	45 (51.1%)	42 (47.7%)	37 (42.1%)	43 (50.6%)
Male	43 (48.9%)	46 (52.3%)	51 (57.9%)	42 (49.4%)
Age group (%)				
≤ 5 years	73 (82.5%)	63 (71.9%)	62 (70.4%)	61 (72.3%)
> 5	15 (17.5%)	25 (28.1%)	26 (29.6%)	24 (27.7%)
Body temperature °C mean (range)	38.2 (36.0–40.6)	37.6 (35.0–40.6)	37.8 (35.1–40.5)	38.1 (35.5–40.7)
Body weight Kg (95% CI)	13.9 (12.8–15.0)	15.3 (14.2v16.5)	16.4 (15.0–17.8)	14.3 (13.3–15.2)
Parasite density, GM (95% CI)	25,770 (19,520–34,021)	28,720 (20,392–40,450)	33,387 (22,888–48,701)	29,850 (20,414–43,648)

*°C* degree Celsius, *Kg* Kilograms, *95% CI* 95% confidence interval. *GM* geometric mean parasite density (asexual parasites/μL), *CI* confidence interval

**Fig. 3:**
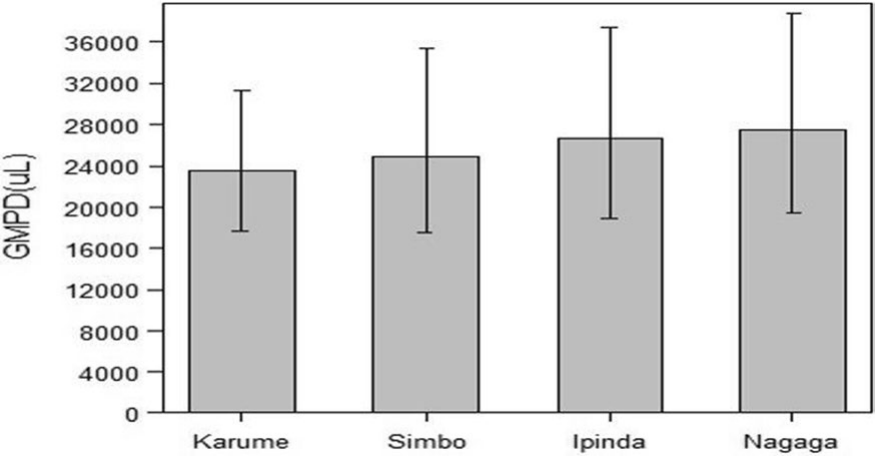
Distribution of geometric mean parasite density between microscopy readers by study site on day 0. Error bars are 95% Confidence intervals

The Karume site recruited more females and children with lower age and body weight compared to other sites. The average axillary temperature was similar at all sites (Table 1). The geometric mean parasite density was comparable across the study sites (Fig. 3).

### Efficacy outcomes

The treatment outcomes are summarized in Table 2. As per the Kaplan–Meier analysis, the PCR-uncorrected ACPR with 95% CI was: 73.9% (63.3–81.8) in Karume, 92% (83.9–96.1) in Ipinda, 80.8% (70.6–87.8) in Simbo, and 90.6% (82.1–95.2) in Nagaga. No early treatment failure was recorded. The PCR results for two patients (one in Ipinda and one in Nagaga) with undetermined results were excluded from PCR corrected analysis. A total of three patients were withdrawn from the study, two from Simbo and one from Ipinda due to protocol violation. The Kaplan–Meier PCR-corrected results showed the ACPR was: 97.7% (91.0–99.4) in Karume, 98.9% (92.2–99.8) in Ipinda, and 100% in Simbo and Nagaga sites. The per protocol PCR-corrected cure rate was 97.0% (88.6–99.3) in Karume, 98.8% (91.5–99) in Ipinda, and 100% in Simbo and Nagaga sites. The recently recommended WHO guidelines on genotyping to detect recrudescent infections after antimalarial treatment (2/3 algorithm)[27] was more sensitive in detecting recrudescent infections resulting in lower cures rates (Additional file 2: Table S2).

**Table 2:** Parasitological and clinical outcomes of enrolled patients

Outcomes	Karume (n = 88)	Simbo (n = 88)	Ipinda (n = 88)	Nagaga (n = 85)
	n (%; 95% CI)	n (%; 95% CI)	n (%), 95% CI)	n (%; 95% CI)
PCR Uncorrected				
ACPR	65(73.9; 63.6–82.1)	67(80.7; 70.7–87.9)	80(92.0;83.8–96.2)	77(90.6;82.1–95.3)
LCF	10(11.4; 6.2–20.0)	6(7.2;3.2–15.3)	2(2.3; 0.6–8.9)	2(2.4; 0.6–9.1)
LPF	13(14.8; 8.7–24.0)	10(12.1; 7–21.1)	5(5.7; 2.4–13.2)	6(7.1; 3.2–15.0)
Excluded				
Lost to follow-up	–	3	–	–
Withdrawn	–	2	1	–
Cumulative cure rate (Kaplan–Meier) (95% CI)	73.9 (63.3–81.8)	80.8 (70.6–87.8)	92.0 (83.9–96.1)	90.6 (82.1–95.2)
PCR corrected				
ACPR	65(97.0; 88.6–99.3)	67(100.0)	80(98.8; 91.5–99.8)	77(100.0)
LCF	0(0)	0(0)	0(0)	0(0)
LPF	2(3.0; 0.7–11.4)	0(0)	1(1.2; 0.2–8.5)	0(0)
Total analysed	67	67	81	77
Excluded				
New Infection	21	16	5	7
Undetermined	0	0	1	1
Loss to follow-up/withdrawn	0	5	1	0
Total excluded	21	21	7	8
Cumulative cure rate (Kaplan–Meier), 95% CI	97.7 (91.0–99.4)	100	98.9 (92.2–99.8)	100

*ETF* Early Treatment Failure, *LPF* Late Parasitological Response, *LCF* Late Clinical Failure, *ACPR* Adequate Clinical and Parasitological Response

### Parasite clearance

**Fig. 4:**
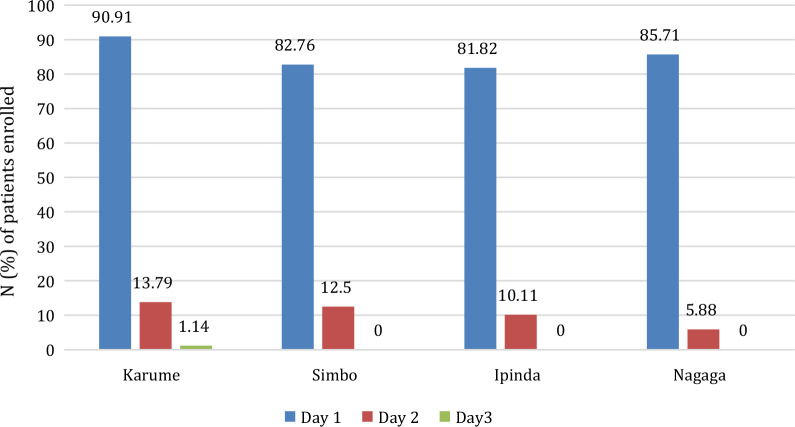
Microscopy positivity among enrolled patients at the four study sites. N (%) of patents enrolled

Karume and Simbo sites had many patients with parasitaemia on day 2 compared to other sites (Fig. 4). One patient from Karume site had parasitaemia on day 3 (272 asexual parasites/μL); however, the parasitaemia was not greater than that of day 0 (11,320 asexual parasites/μL).

### Occurrence of adverse events

A total of 46 adverse events were reported; no serious adverse events (SAE) were recorded. The most common adverse events were cough 24 (52.2%) and skin pallor 11 (23.9%). Distribution of adverse events is shown in Table 3.

**Table 3:** Adverse events reported

Adverse event	Frequencies (n = 46)
Cough	24 (52.2%)
Skin pallor	11 (23.9%)
Painful micturition	3 (6.5%)
Abdominal pain	2 (4.3%)
Dermatitis	1 (2.2%)
Difficulty in breathing	1 (2.2%)
Genital itching	1 (2.2%)
Rectal prolapsed	1 (2.2%)
Vomiting	1 (2.2%)
Wound	1 (2.2%)

### Prevalence of molecular markers of drug resistance before and after treatment with AL

Out of 349 samples collected on day 0, 311 (89.1%) were successfully sequenced for *pfk13* and 328 (94%) were successfully sequenced for *pfmdr1*. Out of 54 samples collected on the day of recurrent infection, 42 (77.8%) were successfully sequenced for *pfk13* and 43 *(79.6%)* were successfully sequenced for *pfmdr1*. Among the 311 sequenced samples collected on day 0, 10 (3.2%) had mutations in the *pfk13* gene; there was no mutation detected on the days of recurrent infection (Table 4). Of the 10 *pfk13* mutations detected on day 0, nine samples had synonymous mutations and one sample had non-synonymous mutation (Table 5). However, these mutations were neither candidate nor validated mutations according to the WHO list published in November 2020 (https://www.who.int/publications/i/item/9789240012813). All successfully sequenced samples on day 0 and on days of recurrent infection were carrying the wild type *pfmdr1* N86. The frequency of parasites carrying mutant type *pfmdr1 184F* increased from 32.9% on day 0 to 55.8% on the days of recurrent infection (Table 4). For *pfmdr1 D1246,* 372/403 (92.3%) of samples were successfully sequenced and all had wild type D1246 polymorphisms (Table 4).

**Table 4:** Frequencies of analysed single-nucleotide polymorphism before treatment on day zero and on days of recurrent infections after treatment with artemether-lumefantrine

Visit day	SNPs (Frequencies, (pure + mix)/total*)			
	*pfk13*	*pfmdr1* N86	*pfmdr1 184F*	*pfmdr1 D1246*
D_0_	10/311 (3.2%)	328/328 (100%)	108/328 (32.9%)	327/327 (100%)
R_0_	0/42 (0%)	43/43 (100%)	24/43 (55.8%)	45/45 (100%)

*D*_*0*_ first day of the study before treatment, *R*_*0*_ Day of recurrent infection, *Total denotes the number of successful analysis

**Table 5:** Polymorphism observed in *pfkelch13* among successfully sequenced samples collected on day of enrollment by site

Site	No. of samples (Day 0)	SNPs (no.)	Codons positions and nitrogenous base (no.)	Type of mutations	Frequency (%)
Ipinda	85	4	C469C (2) P417P (1) R539R (1)	S S S	4.70
Nagaga	88	4	C469C (1) F505F (2) P475S (1)	S S NS	4.82
Karume	79	0	–	–	–
Simbo	94	2	P417P (2)	S	2.47

## Discussion

The study findings show that AL, the recommended first-line ACT for treatment of uncomplicated falciparum malaria in Tanzania, is still highly efficacious, with a PCR-corrected cumulative cure rate ranging from 97% at the Karume site to 100% at the Ipinda and Nagaga sites (per protocol analysis). These results are consistent with those obtained using the Kaplan–Meier method and are similar to our previous findings [5, 19, 23, 28, 29] in the same surveillance areas. Following the 2/3 match method for genotyping *msp1*, *msp2*, and *glurp* genotyping, lower cure rates, as more recrudescent infections were detected; this was similar to previous reports in high transmission settings that observed the main marker leading to discordance between the two analyses was *glurp *[27, 30].

However, the risk of recurrent parasitemia was high in Karume and Simbo, similar to the previous study [20]. The majority of patients with a recurrent infection presented at or after day 21. Potential contributing factors for recurrent infections could be high transmission in the study areas [31] and reduced prophylactic effect after treatment with AL, which is known to have a short elimination half-life.

Artemisinin resistance, defined as partial resistance by the WHO, is phenotypically characterized by prolonged *P. falciparum* clearance time after treatment with an artesunate monotherapy or ACT [32]. Resistance has been linked to specific mutations in the *P. falciparum Kelch 13* propeller gene (*pfk13*) [3, 13, 33]. In this study, 10 *pfk13* mutations were identified, but none were validated or candidate mutations according to the WHO artemisinin resistance protocol [32]. However, mutations have recently been documented in Rwanda, the first report of locally arising *pfk13* mutations in Africa, without affecting the efficacy of AL [15]. From the mutations reported in Rwanda, one was among validated markers (561H) and three were candidate markers (469F, 441L and 449A) [15]. Evidence of partial artemisinin resistance, characterized by delayed parasite clearance with > 10% of patients remaining parasitaemic on day 3 and possessing the kelch13 R561 mutation, has been found in recent research conducted in the Kagera region of Tanzania, which shares a border with Rwanda [17]. Another report from a recent survey in Kibindu-Bagamoyo district show low uncorrected (73.8%) and low PCR-corrected (89.9%) AL efficacy (NMCP unpublished data). Furthermore, a study in Southeast Tanzania found mutations in *k13*-propeller gene, including one sample with R561H, a mutation that has been associated with delayed parasite clearance in Southeast Asia [34].

In this study parasite clearance determined by microscopy remained high, with only one patient having persistent parasitemia of 272 asexual parasites/μL on day 3, similar to other studies in Tanzania [9, 35]. However, there are concerns about the future long-term efficacy of AL in Tanzania, where this artemisinin-based combination has been used as first-line treatment for uncomplicated *P. falciparum* malaria since 2006. Several observations from Bagamoyo district contribute to this concern. High PCR-determined positivity on day 3 after supervised AL treatment in the magnitude of 28–84% has been reported [9, 35].

Widescale use of AL treatment has been associated with selection of wild type alleles (*pfmdr1* N86 and *pfcrt* K76) [10, 36]. In this study only *pfmdr1* polymorphism was analysed. *Pfmdr1* N86 was detected in all successfully sequenced samples collected at enrollment and on the days of recurrent infection. Results from previous study showed higher prevalence of 184F in recurrent infection than in baseline (enrollment) samples [19]. Nonetheless, the presence of mutations does not always correlate with the measured cure rate[37]. However, molecular analysis for *pfmdr1* and *pfcrt* mutation together with *k13* mutations might contribute to understanding the factors underlying causes of tolerance/reduced efficacy [33, 37].

This study also showed that AL was well tolerated with mild adverse events, the most common being cough, skin pallor, and abdominal pain, which were similar to other studies [5, 19, 38]. No serious adverse events were reported in this study. Thorough clinical and laboratory assessment prevented inclusion of patients with suspected severe malaria (severe anemia or parasitemia > 100,000 asexual parasites/µl) or other disease conditions.

This study's limitations included the absence of a fatty meal in conjunction with AL treatment, as advised, potentially impacting lumefantrine absorption. Additionally, differential success in sub-allelic family amplification for msp1, msp2, and glurp might introduce variability in the PCR-corrected outcomes.

## Conclusion

While high cure rates over 97% were observed in these sentinel sites, the notable high rates of reinfection could also indicate that AL is not the best drug to use from the standpoint of preventing spread of resistant parasites. Recent reports of partial artemisinin resistance in Kagera region are of great concern. *Pfmdr1* N86 was detected in all samples and increased selection of *pfmdr1* 184F was observed. These markers have been associated with increased tolerance to AL. Continued surveillance of these markers, along with the markers associated with partial artemisinin resistance, is warranted.

## Disclaimer

The opinions expressed herein are those of the authors and do not necessarily reflect the views of the President’s Malaria Initiative, the U.S. Agency for International Development, the U.S. Centres for Disease Control and Prevention, or other employing organizations or sources of funding. Marian Warsame and Ritha Njau were staff member of the World Health Organization, and they alone are responsible for the views expressed in this publication and do not necessarily represent the decisions, policy or views of the World Health Organization.

### Supplementary Information

**Additional file 1: Table S1.** Locus-by-locus calls and final classification of recrudescence versus new infection

**Additional file 2: Table S2.** PCR corrected cure rates based on 2/3 >= algorithm as recommended by WHO 2021

## Data Availability

No datasets were generated or analysed during the current study.
