# Supplementary material for: Efficacy and safety of artemether-lumefantrine for the treatment of uncomplicated falciparum malaria in mainland Tanzania, 2019
Source: Malar J. 2024 Apr 9;23:101. doi: 10.1186/s12936-024-04931-0 (PMC11005286; doi:10.1186/s12936-024-04931-0)
Supplement: Supplementary file 2 — Additional file 2: Table S2. PCR corrected cure rates based on 2/3 >= algorithm as recommended by WHO 2021 [file 12936_2024_4931_MOESM2_ESM.docx]

Additional file 2: Table S2: PCR corrected cure rates based on 2/3 >= algorithm as recommended by WHO 2021

| Outcomes | Karume (n=88) | Simbo (n=88) | Ipinda (n=88) | Nagaga (n=85) |
| --- | --- | --- | --- | --- |
|  | n (%; 95% CI) | n (%; 95% CI) | n (%; 95% CI) | n (%; 95% CI) |
| PCR corrected  Per protocol analysis |  |  |  |  |
| ACPR | 65 (94.2; 85.3-97.9) | 67(98.5; 90.0-99.8) | 80(97.6; 90.6-99.4) | 77(98.7; 91.2-99.3) |
| LCF | 0(0) | 0(0) | 0(0) | 0(0) |
| LPF | 4(6.0; 2.2-14.7) | 1(1.5;0.2-10.0) | 2(2.4; 0.6-9.4) | 1(01.3; 0.2-8.8) |
| Total analyzed | 69 | 68 | 82 | 78 |
| Excluded |  |  |  |  |
| New Infection | 19 | 15 | 4 | 6 |
| Undetermined | 0 | 0 | 1 | 1 |
| Loss to follow-up/withdrawn | 0 | 5 | 1 | 0 |
| Cumulative cure rate (Kaplan–Meier) | 95.4 (88.2-98.3) | 98.6 (90.6-99.8) | 97.7 (91.1-99.4) | 98.8 (91.9-99.8) |

LCF, late clinical failure; LPF, late parasitological failure; ACPR, adequate clinical and parasitological response.
